# Spreading of pathological TDP-43 along corticospinal tract axons induces ALS-like phenotypes in Atg5^+/-^ mice

**DOI:** 10.7150/ijbs.53872

**Published:** 2021-01-01

**Authors:** Rui Zhang, Yongkang Chen, Xinxin Wang, Haiyan Tian, Han Liu, Zhi Xiang, Dan Qi, Jason H. Huang, Erxi Wu, Xuebing Ding, Xuejing Wang

**Affiliations:** 1 Department of Neurology, Xiangya Hospital, Central South University, Changsha, Hunan, 410008, China; 2 The First Affiliated Hospital of Zhengzhou University, Zhengzhou, Henan, 450052, China; 3 Institute of Parkinson and Movement Disorder, Zhengzhou University, Zhengzhou, Henan, 450052, China; 4 Neuroscience Institute and Department of Neurosurgery, Baylor Scott & White Health, Temple, Texas, 76508, USA; 5 College of Medicine, Texas A&M University, College Station, TX, 77843, USA; 6 Irma Lerma Rangel College of Pharmacy, Texas A&M University, College Station, TX, 77843, USA; 7 LIVESTRONG Cancer Institutes and Department of Oncology, Dell Medical School, the University of Texas at Austin, Austin, TX, 78712, USA; 8 Academy of Medical Sciences, Zhengzhou University, Zhengzhou, Henan, 450052, China

**Keywords:** TAR DNA-binding protein 43 (TDP-43), amyotrophic lateral sclerosis (ALS), autophagy, preformed fibrils (PFFs), Atg5^+/-^ mice

## Abstract

Amyotrophic lateral sclerosis (ALS) is a progressive neurodegenerative disease, characterized by phosphorylated TDP-43 (pTDP-43)-positive inclusions in neurons and glial cells. However, the pathogenic mechanism that underlies ALS remains largely unknown. To investigate the effects of autophagy deficiency in the formation and spreading of pathological TDP-43 along corticospinal tract axons, TDP-43 preformed fibrils (PFFs) were prepared and unilaterally injected into the fifth layer of the left primary motor cortex (M1) or the left anterior horn of the seventh cervical spinal cord segment (C7) of Atg5^+/-^ mice. After the injection of TDP-43 PFFs, the elevated levels of pTDP-43 were present in several pyramidal tract-associated regions of Atg5^+/-^ mice. Additionally, the occurrence of spontaneous potentials detected by electromyography demonstrates evidence of lower motor neuron dysfunction in M1-TDP-43 PFFs-injected Atg5^+/-^ mice, and prolonged central motor conduction time detected by motor evoked potentials provides evidence of upper motor neuron dysfunction in C7-TDP-43 PFFs-injected Atg5^+/-^ mice. These results show that injection of TDP-43 PFFs into the M1 or C7 of Atg5^+/-^ mice induces the spreading of pathological TDP-43 along corticospinal tract axons in both an anterograde and retrograde manner. Importantly, TDP-43 PFFs-injected Atg5^+/-^ mice also display ALS-like motor dysfunction. Taken together, our findings provide direct evidence that TDP-43 PFFs-injected Atg5^+/-^ mice exhibited ALS-like neuropathology and motor phenotypes, suggesting that autophagy deficiency promotes the formation and spreading of pathological TDP-43 *in vivo*.

## Introduction

Amyotrophic lateral sclerosis (ALS) is a progressive neurodegenerative disease 1-4, characterized by phosphorylated TDP-43 (pTDP-43)-positive inclusions in neurons and glial cells 5-7. However, the pathogenic mechanism that underlies ALS remains unclear 8. Postmortem histological studies have identified stereotypical spreading patterns of pTDP-43, and these findings suggest that pTDP-43-positive inclusions may spread along the axons in central nervous system 9, 10. In addition, a few *in vitro* studies suggest that pathological TDP-43 can be transmitted from cell to cell along axon in a self-templating manner 11-16. Furukawa et al. reported that sarkosyl-insoluble TDP-43 fibrils were able to evoke TDP-43 aggregation in HEK293T cells, and this seeding reaction could reproduce ubiquitinated TDP-43 aggregates in cells 12. Furthermore, Nonaka et al. found that sarkosyl-insoluble TDP-43 extracted from ALS or frontotemporal lobar degeneration (FTLD) brains induces TDP-43 aggregation in SH-SY5Y cells, and these cells subsequently formed ubiquitinated, phosphorylated, insoluble cytoplasmic TDP-43 inclusions in a self-templating manner, thus demonstrated seeded aggregation of TDP-43 13. In addition, TDP-43 oligomers may spread intercellularly across axon terminals of primary cortical mouse neurons 14. *In vivo*, by using a zebrafish model, Svahn and his colleagues first demonstrated the nucleo-cytoplasmic transport of TDP-43 17. Until recently, Porta et al. showed that injection of pathological TDP-43 derived from FTLD-TDP brains could lead to the formation and trans-neuronal spreading of TDP-43 pathology in both CamKIIa-hTDP43_NLSm_ mice and non-transgenic mice 18. We speculate that the spreading of pathological TDP-43 via corticospinal tract may be one of the underlying mechanisms of ALS.

In our present study, TDP-43 preformed fibrils (PFFs) were prepared and unilaterally injected into the fifth layer of the left primary motor cortex (M1) or the left anterior horn of the seventh cervical spinal cord segment (C7) of Atg5^+/-^ mice. After the injection of TDP-43 PFFs, the elevated levels of pTDP-43 were present in several pyramidal tract-associated regions of Atg5^+/-^ mice. In addition, the occurrence of spontaneous potentials detected by electromyography (EMG) demonstrates evidence of lower motor neuron (LMN) dysfunction in M1-TDP-43 PFFs-injected Atg5^+/-^ mice, and prolonged central motor conduction time (CMCT) detected by motor evoked potentials (MEPs) suggests upper motor neuron (UMN) lesion in C7-TDP-43 PFFs-injected Atg5^+/-^ mice. Moreover, behavioral analysis indicates that both M1- and C7-TDP-43 PFFs-injected Atg5^+/-^ mice developed an ALS-like syndrome in a time-dependent manner.

## Materials and methods

### Animals

Eight-week-old male B6: 129-Atg5<tm1Nmz> (RBRC02231) (Atg5^+/-^) and C57BL/6 mice weighing 18-20 g were used. Animals were kept under standard laboratory conditions with free access to standard laboratory food and water (21 °C, 12h/12h light-dark cycle). All experiments were performed in accordance with the Guide for the Care and Use of Laboratory Animals. The protocols were approved by the Institutional Ethics Committees of the Zhengzhou University.

### TDP-43 PFFs preparation and Electron Microscopy Imaging

TDP-43 PFFs were prepared as previously reported 11, 12. Monomeric TDP-43 (Proteintech, Wuhan, China) was resuspended in sterile water at concentration of 0.5 mg/ml. To obtain TDP-43 PFFs, the samples were incubated at 37 ºC for 2 hours with agitation at 600 rpm in an Eppendorf Thermomixer. For electron microscopy imaging, a 5 µl sample was absorbed onto carbon/formvar-coated 150 mesh copper grids (Yasheng Electronics Technology Co, Ltd., Zhengzhou, China) for 30 seconds and then stained with 5 µl of 2% uranyl acetate for 30 seconds. After staining, the grids were rinsed briefly in distilled water. The excess water was removed with filter paper, and the grids were air-dried prior to analysis by transmission electron microscopy (JEOL USA, Inc., Peabody, MA, USA).

### Stereotaxic surgery on mice

One hundred and twenty Atg5^+/-^ mice were divided into 4 groups equally: M1-TDP-43 PFFs injection group, M1-PBS injection group, C7-TDP-43 PFFs injection group, and C7-PBS injection group. The mice were deeply anesthetized using isoflurane during the whole surgical procedure and immobilized in a stereotaxic frame (David Kopf Instruments, Tujunga, CA, USA). Unilateral injections were made into the fifth layer of the left M1 or left anterior horn of C7 at the following coordinates: M1: anteroposterior, 0.7 mm; mediolateral, 1.5 mm; dorsoventral, 1.4 mm; and C7: mediolateral, 0.8 mm; dorsoventral, 1.2 mm. Each injection site received 5 µl of TDP-43 PFFs or PBS at a rate of 0.2 µl/minute using a Hamilton Syringe (Hamilton, NV, USA).

### Survival analysis

Date and cause of death were recorded for each mouse. For ethical reasons, animals were closely monitored and sacrificed as moribund prior to actual death using criteria for severe illness. To determine the duration of survival reliably and humanely, the moribund state, defined as the inability of mice to right themselves 30 seconds after being placed on their side, was used. The moribund mice were scored as “dead” and were euthanized.

### Immunohistochemistry

After euthanasia and perfusion, the brain and spinal cord from perfused animals were fixed in 30% sucrose solution containing 4% paraformaldehyde. Tissues were embedded in paraffin blocks, cut into 4 µm using a Rotary Microtome (Leica RM2235, Leica, Nussloch, Germany), and mounted on glass slides. Sections of spinal cord and brain tissues were then deparaffinized and rehydrated in a graded ethanol series. Immunohistochemistry was performed as previously described 19. Briefly, tissues were incubated in 3% hydrogen peroxide solution and then subjected to antigen retrieval at 100 °C for 10 minutes in citrate buffer. Tissues were blocked in normal goat serum for 20 minutes at room temperature and then incubated with anti-pTDP-43 (phosphorylated at Ser409/Ser410) antibody (mouse, Millipore, 1:800) at 4 °C for 24 hours. Then, the labeling was detected using a Streptavidin-Peroxidase kit (Bioss, China) and visualized with diaminobenzidine. Images were captured using an Olympus IX51 microscope mounted with a DP71 Olympus digital camera.

### Preparation of mouse tissue lysates and immunoblotting

Frozen spinal cord or brain samples were thawed on ice and sonicated in 5× v/w RIPA buffer (50 mM Tris, 150 mM NaCl, 1% NP-40, 5 mM EDTA, 0.5% sodium deoxycholate, and 0.1% SDS, pH 8.0) containing protease inhibitor cocktails (Thermo Fisher Scientific). Samples were centrifuged for 30 minutes at 4 °C, 100,000 g. The supernatant was taken as RIPA-soluble fractions. The pellet was sonicated with 5× v/w RIPA buffer as above. Then, the pellet was sonicated in 2× v/w urea buffer (7 M urea, 2 M thiourea, 4% CHAPS, and 30 mM Tris, pH 8.5) and centrifuged for 30 minutes at 22 °C, 100,000 g. This supernatant was collected as the RIPA-insoluble/urea-soluble fractions. Samples were analyzed by 10% SDS-PAGE with polyvinylidene fluoride (PVDF) membranes. Following transfer to the PVDF membranes, the immunoblot was sequentially probed with antibodies that recognize pTDP-43 (mouse, Millipore, 1:800) or LC3A/B (rabbit, Cell Signaling Technology, 1:1000). The densities of the proteins were normalized to GAPDH.

### Needle EMGs

Mice were anesthetized with isoflurane and placed in the prone position on a thermostatically controlled warming plate to maintain body temperature at 37 ^°^C. A standard EMG apparatus (MEB-2306C, Nihon Kohden Corporation, Tokyo, Japan) was applied. To determine UMN and LMN deficits, we collected motor unit action potentials (MUAPs) and spontaneous activities including fibrillation potentials, positive sharp waves and fasciculation potentials. A sweep speed of 10 ms/div, a gain of 100 uv/div and a band pass filter with low and high cut-off frequency of 20 and 10,000 Hz were performed. The mice were electrically grounded through a disposable needle electrode implanted subcutaneously at the abdominal wall. Next, we used concentric needles (25.0 mm × 0.3 mm, Technomed Europe, Beek, Netherlands) to detect the following muscles: bilateral biceps brachii, bilateral T10 paraspinal muscles, bilateral tibialis anterior, and bilateral gastrocnemius. Spontaneous activities were recorded for 120 seconds and MUAPs were recorded at different sites of each muscle until 20 MUAPs were obtained.

### Electrical stimulation and recordings of MEPs

Cortical MEP (cMEP) and spinal MEP (sMEP) were obtained using two monopolar needle electrodes. For cMEP, the cathode was positioned at the midline of the interaural line through the scalp; the anode was positioned 4-5 mm lateral and anterior to the cathode. For sMEP, two monopolar needle electrodes were inserted into C7, close to the emergence of the spinal nerve root. cMEP and sMEP were recorded with the active needle electrode inserted into the muscle of the forelimb footpad and reference needle electrode inserted under the skin of the second digit. The onset of the first, generally negative, deflection was taken as the MEP latency. The MEP with the shortest latency was considered as the final evaluation criteria of MEP latency, the peak-to-peak amplitude of the motor response was used for the mean MEP amplitude. The CMCT is the propagation time between motor cortex and spinal cord, which is the difference between the cMEP latency and the sMEP latency.

### H&E staining

Biopsied biceps brachii were dissected out from the forelimb of an anesthetized animal and immersed immediately in isopentane cooled in liquid nitrogen. Serial frozen sections were cut at 10 μm thickness and stained by hematoxylin and eosin (H&E). All steps were performed according to the standard procedures 20.

### Behavioral tests

To evaluate behavioral performance, 15 mice from each group were weighed weekly and underwent a battery of behavioral tests every two weeks starting from the second month post-surgery. Baseline performance was measured before surgery. The experimenter was blinded to the treatment group for all behavioral tests.

#### Rotarod test

Rotarod test was performed using the rotarod apparatus (Rotarod YLS-4C; YiYan Science and Technology Development Co., Ltd. Shandong, China). The test began by placing the mice on the rod rotating at 30 rpm. The latency to fall off the rotarod within the maximum time (180 seconds) was recorded as a measurement of the competence of their motor function. Three trials were performed per day with a 15-minute inter-trial interval. The mean latency to fall off the rotarod was calculated for the data analysis.

#### Hanging wire test

The hanging wire test was performed to evaluate general muscle strength. Each mouse was placed on a horizontally positioned screen with many 1 cm × 1 cm grids. The period until the mouse left the screen was recorded, with a maximum time of 180 seconds. Three trials were performed per day, and the mean latency was recorded for the data analysis.

#### Footprint test

To obtain footprints, paws of the mice were painted with two different non-toxic paints (fore-paws in red and hind-paws in green). The mice were then allowed to walk along a restricted cardboard tunnel (50 cm long, 5 cm wide, 10 cm high) into an enclosed box and a sheet of white paper was placed on the floor of the tunnel, and one set of footprints were collected for each mouse. Three steps from the middle portion of each run were measured for the following parameters (cm): (1) stride length (front and hind legs). (2) The front- and hind-base width. The mean value of each set of values was used for the data analysis.

### Statistical analysis

The statistical analyses were performed using SPSS 24.0 (IBM, Armonk, New York, USA). Behavioral and electrophysiological data of mice were presented as mean ± standard deviation (SD), and two-way ANOVA was employed for comparison of multiple groups. Disease onset and survival statistics were performed by Kaplan-Meier survival curves and the data were analyzed using the log-rank test, generating a χ^2^ value to test for significance. For all statistical tests, significance was taken as *p < 0.05, **p < 0.01, ***p < 0.001.

## Results

### TDP-43 PFFs induce pTDP-43-immunoreactive deposits *in situ*

**Figure 1:**
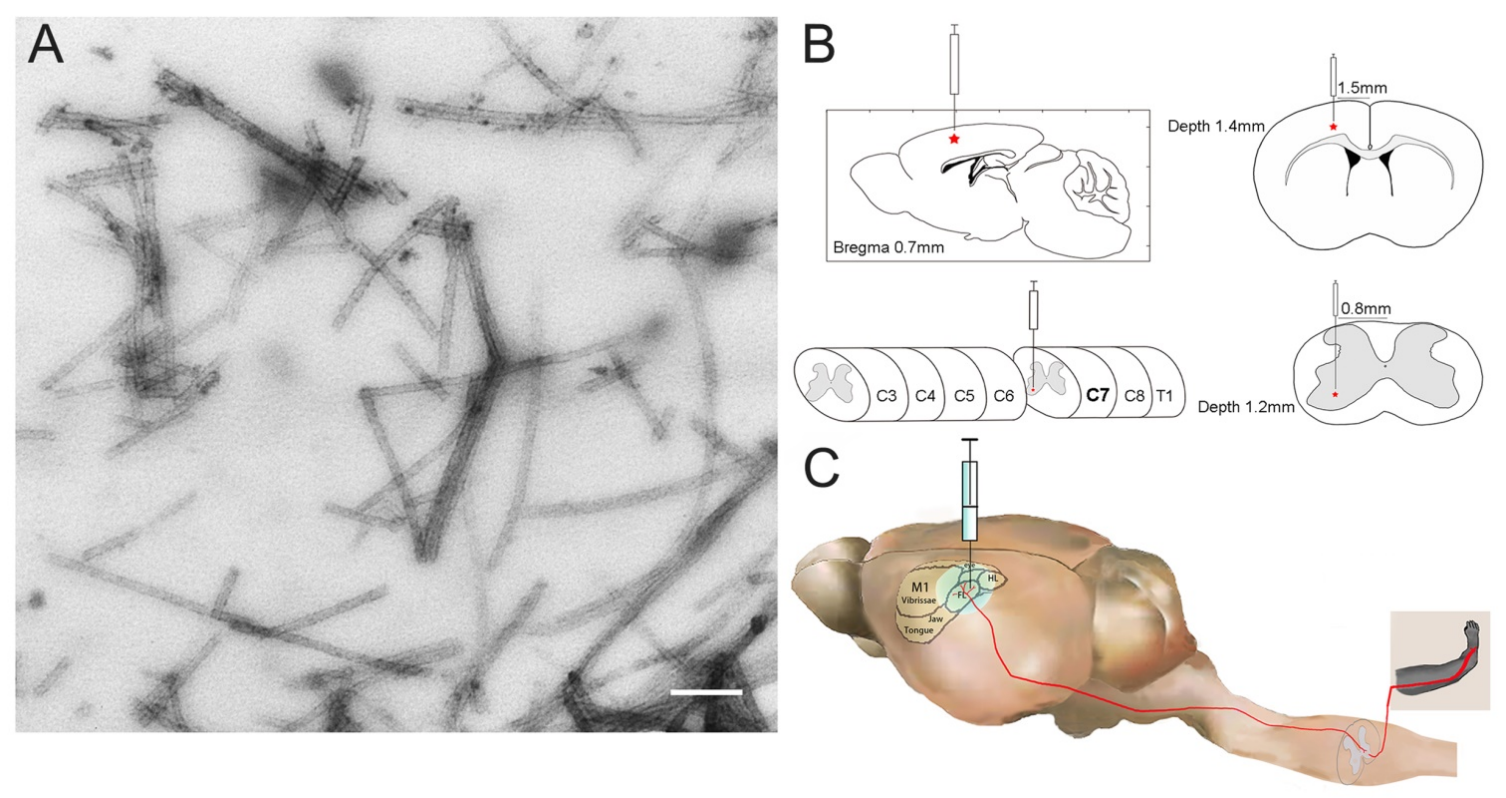
** The construction of M1- and C7-TDP-43 PFFs-injected Atg5^+/-^ mouse model. A.** Negatively stained transmission electron micrograph of TDP-43 PFFs. Scale bar = 200 nm. **B.** The schematic diagram of M1- and C7-TDP-43 PFFs/PBS-injected Atg5^+/-^ mouse model in the coronal and axial position. **C.** The nerve conduction pathway from the forelimb region of M1 to the skeletal muscles of the forelimb.

**Figure 2:**
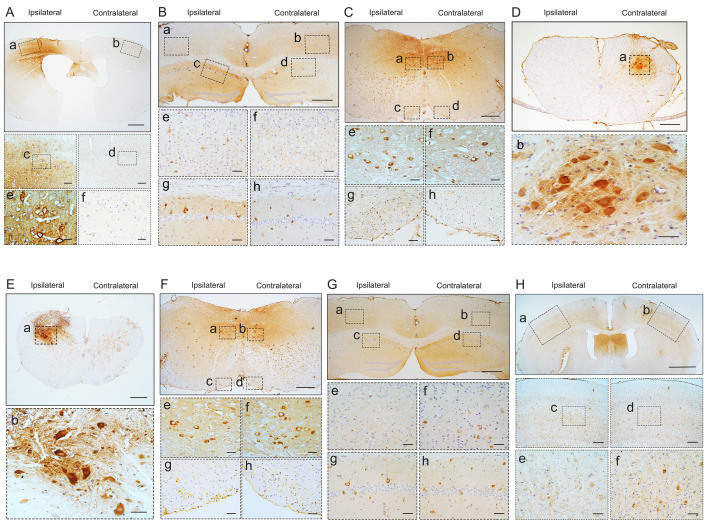
**Pathological TDP-43 spread along corticospinal tract axons anterogradely and retrogradely.** pTDP-43 staining was performed in M1 (A) at 2 mpi, hippocampus (B), medulla oblongata (C), and cervical enlargement (D) at 6 mpi of M1-TDP-43 PFFs-injected Atg5^+/-^ mice. **A.** In the second, third and fifth layer of the M1, pTDP-43-ir in neurons and glial cells was more abundant in the left side (a, c, and e) than the contralateral side (b, d, and f). Scale bars: a-b, 1000 μm; c-d, 100 μm; e-f, 25 μm. **B.** In the fifth layer of M1 and CA1 region of hippocampus, neuronal and glial cytoplasmic pTDP-43-ir was also more severe in the left side (a, c, e, and g) than contralateral side (b, d, f, and h). Scale bars: a-d, 1000 μm; e-h, 100 μm. **C.** In medulla oblongata, neuronal and glial cytoplasmic pTDP-43-ir was stronger in the left side of the hypoglossal nucleus (a and e) and pyramidal tract (c and g) than the contralateral side (b, f, d and h). Scale bars: a-d, 500 μm; e-h, 50 μm. **D.** In cervical enlargement, neuronal and glial cytoplasmic pTDP-43-ir was mainly deposited in the right side of the anterior horn (a and b). Scale bars: a, 500 μm; b, 50 μm. pTDP-43 staining was performed in cervical enlargement (E) at 2 mpi, medulla oblongata (F), hippocampus (G), and M1 (H) at 5 mpi of C7-TDP-43 PFFs-injected Atg5^+/-^ mice. **E.** In cervical enlargement, pTDP-43-ir was predominantly deposited in the neurons of the left anterior horn (a and b). Scale bars: a, 500 μm; b, 50 μm. **F.** In medulla oblongata, neuronal and glial cytoplasmic pTDP-43-ir was stronger in the right side of hypoglossal nucleus (b and f) and py (d and h) than left side (a, c, e and g). Scale bars: a-d, 500 μm; e-h, 50 μm. **G.** In the fifth layer of the M1 and CA1 region of the hippocampus, neuronal and glial cytoplasmic pTDP-43-ir was also more severe in the right side (b, d, f, and h) than left side (a, c, e, and g). Scale bars: a-d, 1000 μm; e-h, 100 μm. **H.** In the fifth layer of M1, neuronal and glial cytoplasmic pTDP-43-ir was more abundant in the right side (b, d, and f) than left side (a, c, and e). Scale bars: a-b, 1000 μm; c-d, 100 μm; e-f, 25 μm.

**Figure 3:**
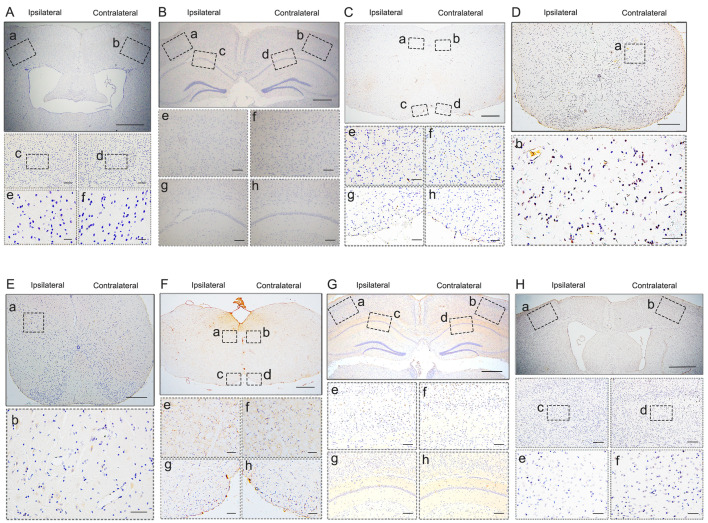
** pTDP-43 staining in M1- and C7-PBS-injected Atg5^+/-^ mice. A-H.** No pTDP-43 pathology was found in both sides of M1- and C7-PBS-injected Atg5^+/-^ mice (compared with Figure 2).

To investigate whether pathological TDP-43 could spread along corticospinal tract axons, we injected TDP-43 PFFs (**Figure 1A**) into the fifth layer of the left M1 or the left anterior horn of C7 of Atg5^+/-^ mice (**Figure 1B**). Diagrams of estimated conduction pathway for pathological TDP-43 spreading were shown in **Figure 1C**. Firstly, pTDP-43 immunoactivity was detected at the injection sites using an anti-pTDP-43 (phosphorylated at Ser409/Ser410) antibody. As early as 2 months post-injection (mpi), pTDP-43-immunoreactive (pTDP-43-ir) pathology was detected in the neurons and glial cells in the second, third and fifth layers of the M1 around the injection region of M1-TDP-43 PFFs-injected Atg5^+/-^ mice (**Figure 2Aa, 2Ac,** and** 2Ae**); however, the staining was not observed on the contralateral side (**Figure 2Ab, 2Ad,** and** 2Af**). In the cervical enlargement of C7-TDP-43 PFFs-injected Atg5^+/-^ mice, pTDP-43-ir pathology was found in the neurons of the left spinal cord anterior horn adjacent to the injection sites at 2 mpi (**Figure 2Ea** and** 2Eb**). No pTDP-43-ir pathology was detected in the age-matched PBS-injected Atg5^+/-^ mice (**Figure 3A** and **3E**). Thus, these data indicated that TDP-43 PFFs could induce the formation of pTDP-43 pathology *in vivo*. These data supported that the injection of TDP-43 PFFs into the brain or spinal cord induce pTDP-43-ir pathology deposits in situ.

### Pathological TDP-43 spreads along corticospinal tract axons in both anterograde and retrograde manner

To demonstrate whether spreading of pathological TDP-43 via corticospinal tract induces ALS-like neuropathology, we next evaluated the pTDP-43 pathology in different sections along the corticospinal tract at different time points in Atg5^+/-^ mice.

At about 6 mpi, pTDP-43-ir staining was detected in the hippocampus (**Figure 2B**), medulla oblongata (**Figure 2C**), and cervical enlargement (**Figure 2D**) of M1-TDP-43 PFFs-injected Atg5^+/-^ mice. In the cortex, neuronal and glial cytoplasmic pTDP-43-ir pathology mainly deposited in the fifth layer of the left M1 (**Figure 2Ba** and** 2Be**), while no pTDP-43 pathology was observed on the contralateral side (**Figure 2Bb** and** 2Bf**). In the CA1 region of the hippocampus, the positive neuronal and glial cytoplasmic pTDP-43 staining was more intense on the left side (**Figure 2Bc** and** 2Bg**) than the contralateral side (**Figure 2Bd** and** 2Bh**). In medulla oblongata, the positive neuronal and glial cytoplasmic pTDP-43 pathology was detected in both sides of hypoglossal nucleus (**Figure 2Ca, 2Cb, 2Ce,** and** 2Cf**) and the pTDP-43-ir nerve fibers in the left corticospinal tract (**Figure 2Cc** and** 2Cg**), and the pTDP-43 staining was stronger in the left side of the hypoglossal nucleus (**Figure 2Ca** and **2Ce**), however, the pTDP-43-ir staining was not detected in the contralateral side of the corticospinal tract (**Figure 2Cd** and** 2Ch**). Furthermore, neuronal and glial cytoplasmic pTDP-43 was deposited in the right side of anterior horn of the cervical enlargement (**Figure 2Da** and** 2Db**). However, no pTDP-43 pathology was detected in both sides of M1-PBS-injected Atg5^+/-^ mice (**Figure 3B-D**).

**Figure 4:**
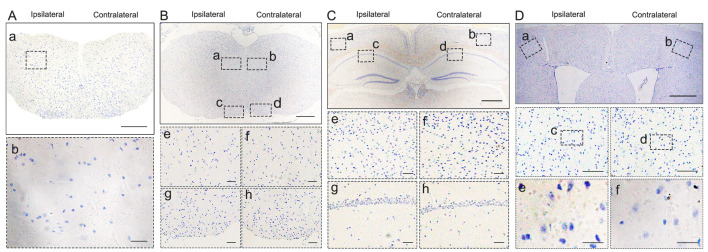
**pTDP-43 staining in C7-TDP-43 PFFs-injected C57BL/6 mice. A-D.** No pTDP-43 pathology was found in both sides of C7-TDP-43 PFFs-injected Atg5^+/-^ at 18 mpi (compared with **Figure 2E-H**).

In C7-TDP-43 PFFs-injected Atg5^+/-^ mice, neuronal and glial cytoplasmic pTDP-43 staining was more frequently observed in the right side of the hypoglossal nucleus (**Figure 2Fb** and** 2Ff**), CA1 region of hippocampus (**Figure 2Gd** and** 2Gh**), and the fifth layer of the M1 (**Figure 2Gb, 2Gf, 2Hb, 2Hd,** and** 2Hf**) compared to the left side (**Figure 2Fa, 2Fc, 2Fe, 2Fg, 2Ga, 2Gc, 2Ge, 2Gg, 2Ha, 2Hc,** and** 2He**) at about 5 mpi. The pTDP-43-ir nerve fibers were detected in the left corticospinal tract at about 5 mpi (**Figure 2Fd** and** 2Fh**). As with M1-PBS-injected Atg5^+/-^ mice, no pTDP-43 pathology was detected in both sides of C7-PBS-injected Atg5^+/-^ mice (**Figure 3F-H**). In addition, we also injected TDP-43 PFFs into the C7 of C57BL/6 mice. However, the formation of TDP-43-ir neuronal and glial cytoplasmic pathology in the fifth layer of the M1 was not detected until 18 months (**Figure 4**). Taken together, these data suggest that the injection of TDP-43 PFFs into motor cortex or ventricornu of Atg5^+/-^ mice induces the spreading of TDP-43 pathology along corticospinal tract axons anterogradely and retrogradely *in vivo*.

**Figure 5:**
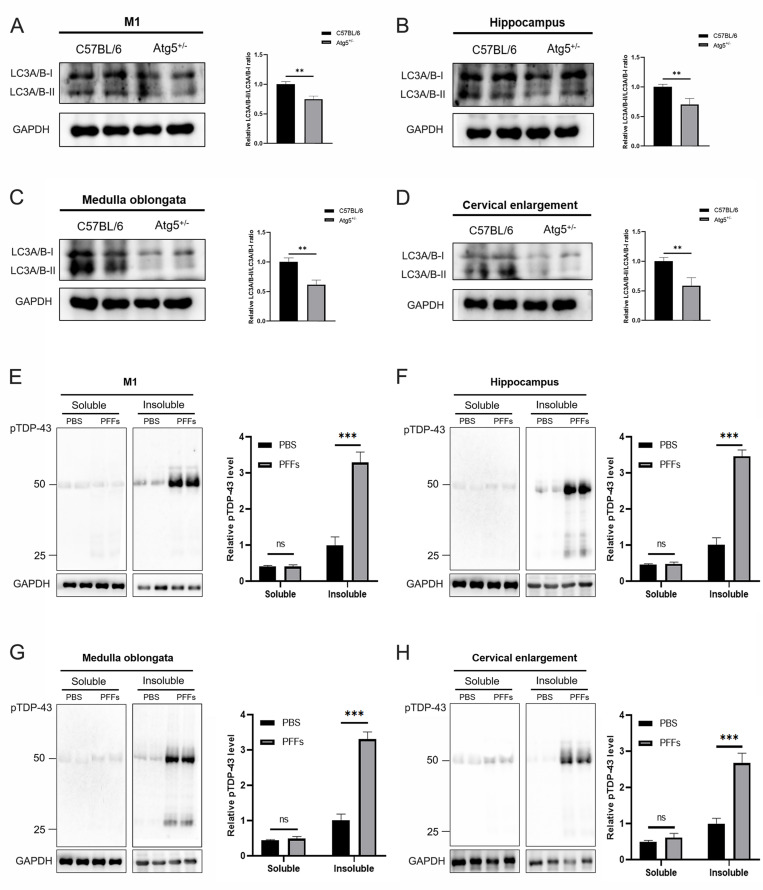
** Immunoblotting analysis of LC3A/B and pTDP-43. A-D.** Representative immunoblotting of LC3A/B in M1 (A), hippocampus (B), medulla oblongata (C), and cervical enlargement (D) of adult Atg5^+/-^ mice and age-matched C57BL/6 mice. **E-H.** Representative immunoblotting of pTDP-43 in the RIPA-soluble and insoluble fractions of M1 (E), hippocampus (F), medulla oblongata (G), and cervical enlargement (H) of M1-TDP-43 PFFs-injected Atg5^+/-^ mice (at 6 mpi) and age-matched M1-PBS- injected Atg5^+/-^ mice. Blots were probed for GAPDH as loading control (bottom).

To evaluate whether autophagy deficiency can affect the aggregation of pathological TDP-43, we first detected LC3 expression levels in M1, hippocampus, medulla oblongata, and cervical enlargement of C57BL/6 and Atg5^+/-^ mice, the results showed that LC3A/B-II/LC3A/B-I levels were significantly decreased in these regions of adult Atg5^+/-^ mice compared with age-matched C57BL/6 mice (**Figure 5A-D**). To further demonstrate whether there was an increase of pTDP-43 pathology in TDP-43 PFFs-injected Atg5^+/-^ mice, immunoblotting analysis was also performed in the abovementioned regions of M1-TDP-43 PFFs- and M1-PBS-injected Atg5^+/-^ mice at 6 mpi. In the soluble fractions, no significant difference of pTDP-43 levels was found between M1-TDP-43 PFFs- and M1-PBS-injected Atg5^+/-^ mice (**Figure 5E-H**). Nevertheless, in the insoluble fractions, pTDP-43 levels were increased in all examined M1-TDP-43 PFFs-injected Atg5^+/-^ mice, while it was faintly detected in age-matched PBS-injected Atg5^+/-^ mice (**Figure 5E-H**). The same results were also found in C7-TDP-43 PFFs-injected Atg5^+/-^ mice at 5 mpi (data not shown). These results support that the injection of TDP-43 PFFs into M1 or C7 of Atg5^+/-^ mice could induce the spreading of pathological TDP-43 along corticospinal tract axons anterogradely and retrogradely.

### M1- and C7-TDP-43 PFFs-injected Atg5^+/-^ mice exhibit ALS-like neurophysiological phenotypes

**Figure 6:**
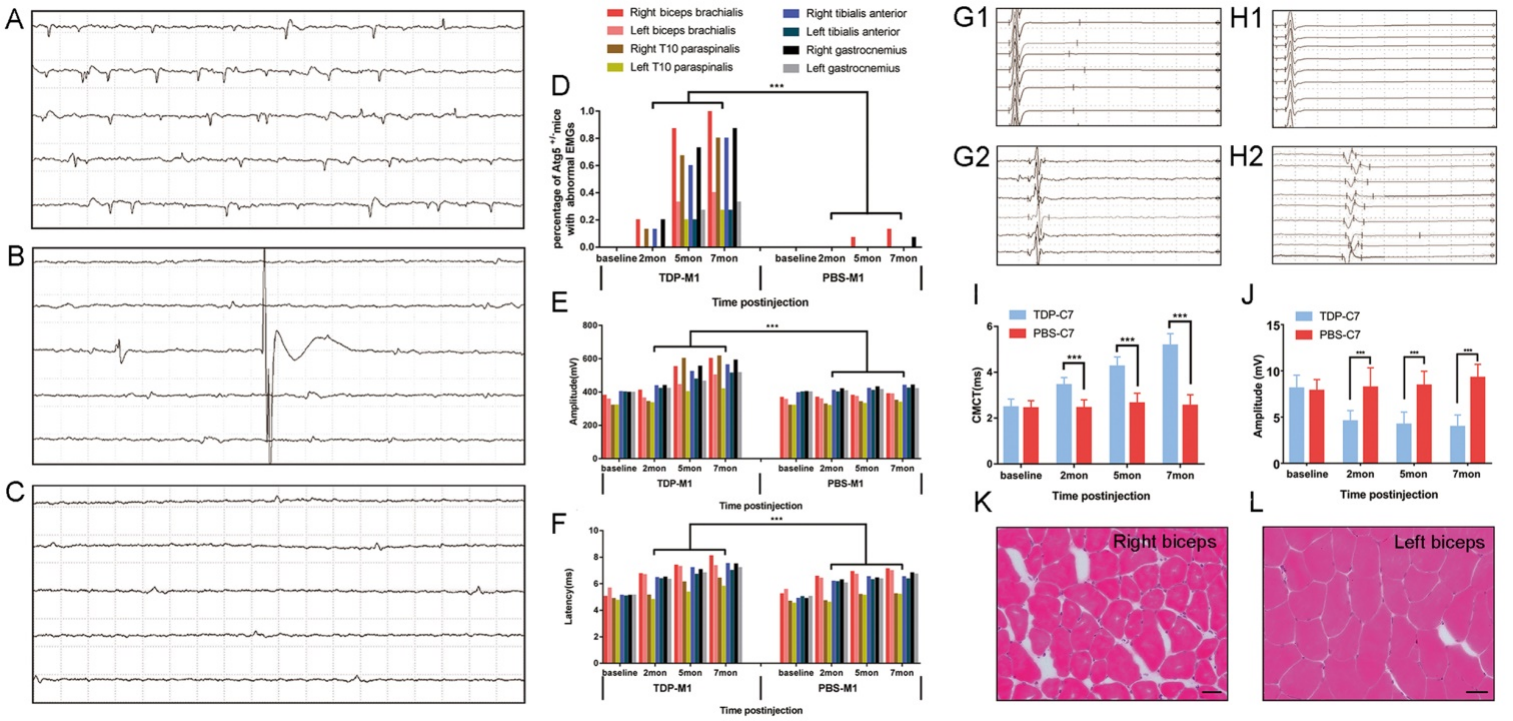
** TDP-43 PFFs-injected Atg5^+/-^ mice developed ALS-like changes in neurophysiology and skeletal muscle morphology. A-C.** At 2 mpi, spontaneous potentials including fibrillation potentials (A), fasciculation potentials (B), and positive sharp waves (C) were detected in M1-TDP-43 PFFs-injected Atg5^+/-^ mice. **D.** The frequencies of spontaneous potentials were much higher in M1-TDP-43 PFFs-injected mice compared to M1-PBS-injected Atg5^+/-^ mice. **E-F.** The amplitude (E) and latency of MUAPs (F) were increased in M1-TDP-43 PFFs-injected mice compared to M1-PBS-injected Atg5^+/-^ mice, *p* < 0.05. **G-H.** Left-sMEP (L-sMEP) (G1 and H1) and Right-cMEP (R-cMEP) (G2 and H2) were detected in C7-PBS-injected mice (G1 and G2) and C7-TDP-43 PFFs-injected mice (H1 and H2). **I.** The CMCT was increased in C7-TDP-43 PFFs-injected mice compared to C7-PBS-injected mice at different time points post injection. **J.** The amplitude of cMEP was decreased in C7-TDP-43 PFFs-injected Atg5^+/-^ mice compared to M1-PBS-injected Atg5^+/-^ mice at different time points post injection. **K-L.** At the end-stage of M1-TDP-43 PFFs-injected Atg5^+/-^ mice, H&E staining of right biceps (K) showed muscle atrophy, round muscle fibers, and muscle fibers with central nuclei, while the left biceps (L) showed normal skeletal muscle morphology. Scale bar = 25 μm.

Besides the morphological evidence of TDP-43 PFFs-induced pathology, we further investigated whether the M1- and C7-TDP-43 PFFs-injected Atg5^+/-^ mice exhibited any neurophysiological abnormality. Spontaneous activity, motor unit potential recruitment, and MUAPs were used to quantify LMN dysfunction. MEPs were used to evaluate potential impairment of the corticospinal tract. Firstly, needle EMGs were recorded from the biceps brachialis, T10 paraspinals, tibialis anterior, and gastrocnemius muscles. In M1-TDP-43 PFFs-injected mice, abnormal spontaneous activity including fibrillation potentials (**Figure 6A**), fasciculation potentials (**Figure 6B**), and positive sharp waves (**Figure 6C**) were detected on the right side of biceps brachialis, T10 paraspinals, tibialis anterior, and gastrocnemius muscles as early as 2 mpi. At 5 mpi, abnormal spontaneous activities were found in both side of biceps brachialis, T10 paraspinals, tibialis anterior, and gastrocnemius muscles, and there was no significantly difference between the two sides (**Figure 6D**). Significantly, the abnormal spontaneous activities were detected in the right biceps brachialis of all the M1-TDP-43 PFFs-injected mice, however, the M1-PBS-injected Atg5^+/-^ mice only showed mild abnormal spontaneous activity in right biceps brachialis muscles at 7 mpi (**Figure 6D**). The frequency of abnormal spontaneous activity was developed in a time-dependent manner as shown in **Figure 6D**. Secondly, we collected 20 MUAPs in each mouse for the motor unit measurement. The findings showed a significantly increase in the mean amplitude and duration of MUAPs at 2, 5, and 7 mpi in M1-TDP-43 PFFs-injected Atg5^+/-^ mice, compared to age-matched M1-PBS-injected Atg5^+/-^ mice (**Figure 6E** and** 6F**). Furthermore, we performed a biopsy in both sides of the biceps brachialis in M1-TDP-43 PFFs-injected Atg5^+/-^ mice at 7 mpi. H&E staining showed muscle atrophy, round muscle fibers, and muscle fibers with central nuclei in the right biceps brachialis** (Figure 6K),** while no morphological changes were found in the left biceps brachialis (**Figure 6L**). Taken together, these findings suggest LMN dysfunction in M1-TDP-43 PFFs-injected Atg5^+/-^ mice.

Furthermore, we performed cMEP and sMEP in C7-TDP-43 PFFs-injected Atg5^+/-^ mice to quantify UMN impairment (**Figure 6G1, 6G2, 6H1** and** 6H2**). At about 2 mpi, CMCT was significantly prolonged in the injection side of C7-TDP-43 PFFs-injected Atg5^+/-^ mice compared to age-matched C7-PBS-injected Atg5^+/-^ mice (3.1 ± 0.35 ms versus 2.44 ± 0.36 ms, *p* < 0.05), and the CMCT increased in a time-dependent manner (**Figure 6I**) in C7-TDP-43 PFFs-injected Atg5^+/-^ mice. Moreover, cMEP amplitude decreased in C7-TDP-43 PFFs-injected Atg5^+/-^ mice compared to age-matched C7-PBS-injected Atg5^+/-^ mice (4.57 ± 1.14 mV versus 8.23 ± 2.10 mV, *p* < 0.001) at about 2 mpi, and this decline became more significant over time (**Figure 6J**). At the end stage, responses to cortical stimulation were absent in C7-TDP-43 PFFs-injected Atg5^+/-^ mice, indicating devastating damage of the corticospinal tract. Taken together, the quantitative analyses of CMCT and cMEP amplitude indicated UMN dysfunction in C7-TDP-43 PFFs-injected Atg5^+/-^ mice.

### M1- and C7-TDP-43 PFFs-injected Atg5^+/-^ mice exhibit motor impairments

**Figure 7:**
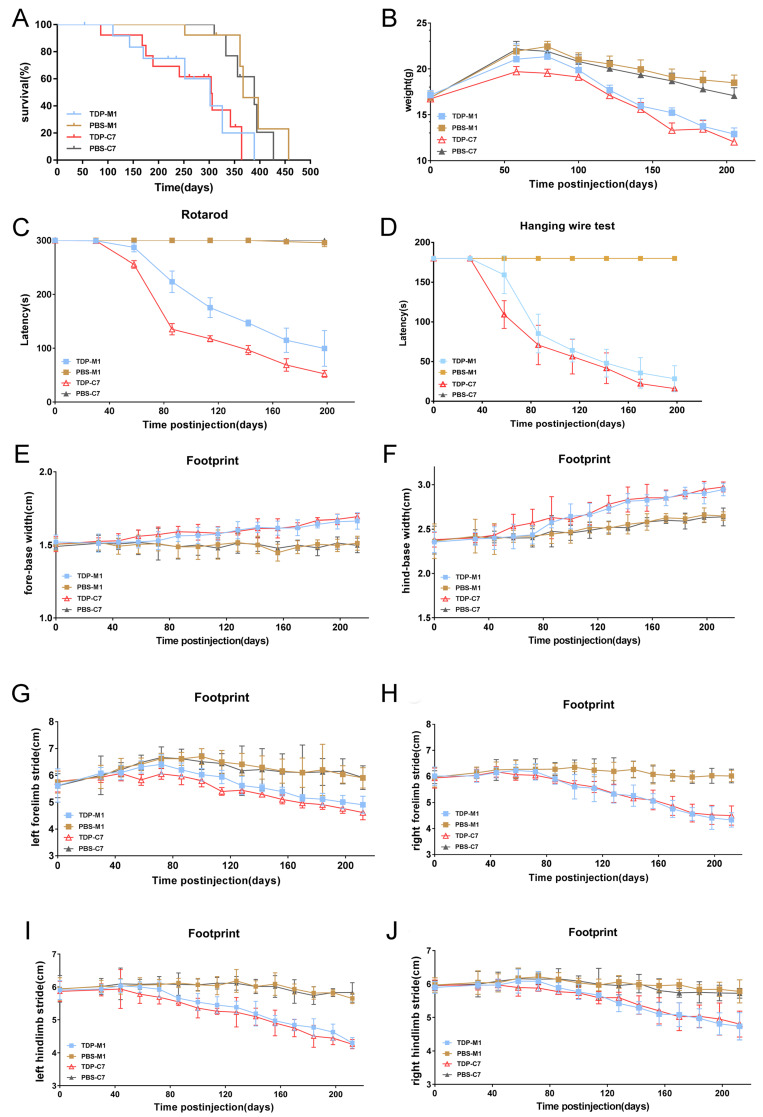
** M1- and C7-TDP-43 PFFs-injected Atg5^+/-^ mice developed motor impairments. A.** Kaplan-Meier survival probability curve showed that the life span of TDP-43 PFFs-injected Atg5^+/-^ mice was shorter than PBS-injected Atg5^+/-^ mice. **B.** M1- and C7-TDP-43 PFFs-injected Atg5^+/-^ mice developed meaningful weight decline at 8 weeks post injection compared to M1- and C7-PBS-injected mice, *p* < 0.001. **C.** Rotarod test analysis of mice at various time post injection revealed that C7-TDP-43 PFFs-injected Atg5^+/-^ mice had significant differences in rotarod latencies at 8 weeks post injection, M1-TDP-43 PFFs-injected Atg5^+/-^ mice at 10 weeks compared to age-matched PBS-injected controls, *p* < 0.001. **D.** Hanging wire test of mice at various times post injection revealed that C7-TDP-43 PFFs-injected Atg5^+/-^ mice had significant differences in hanging wire latencies at 8 weeks post injection, M1-TDP-43 PFFs-injected Atg5^+/-^ mice at 10 weeks compared to age-matched PBS-injected controls, *p* < 0.001. Longer latency indicates greater strength. **E-J.** The footprint test was performed on Atg5^+/-^ mice every two weeks from 30 days to 200 days. C7-TDP-43 PFFs-injected mice showed shorter stride length (E-H) and wider base width (I and J) compared to C7-PBS-injected controls at 8 weeks post injection, *p* < 0.001. M1-TDP-43 PFFs-injected Atg5^+/-^ mice performed meaningful changes at 10 weeks compared to age-matched PBS-injected controls. A two-way ANOVA followed by Bonferroni adjustment was used for statistical analysis. Data represent means ± SD of four independent trials (n = 15 mice/group).

We further investigated whether M1- and C7-TDP-43 PFFs-injected Atg5^+/-^ mice exhibit ALS-like symptoms. First, the lifespan of TDP-43 PFFs-injected Atg5^+/-^ mice was significantly shorter compared with PBS-injected controls, and the Kaplan-Meier curve showed overall survival in** Figure 7A**. To assess the motor function of TDP-43 PFFs-injected Atg5^+/-^ mice, we performed a series of behavioral tests. At approximately 8 weeks post-injection, C7-TDP-43 PFFs-injected Atg5**^+/-^**mice gradually lost weight (**Figure 7B**) and performed poorly on the rotarod test (**Figure 7C**) and hanging wire test (**Figure 7D**), revealing a significant deficit in muscle strength, motor coordination, and balance. In addition, C7-TDP-43 PFFs-injected Atg5**^+/-^**mice showed significantly shorter stride length and wider base width compared to PBS-injected controls in the footprint test (**Figure 6E-J**). As for M1-TDP-43 PFFs-injected Atg5**^+/-^**mice, they began to lose weight at around 10 weeks post-injection (**Figure 7B**), and they also showed deficient motor performance in the rotarod test and hanging wire test compared to age-matched PBS-injected Atg5**^+/-^**mice (**Figure 7C** and** 7D**). However, the severity of motor disability was much higher in C7-TDP-43 PFFs-injected Atg5**^+/-^**mice compared to age-matched C7-TDP-43 PFFs-injected Atg5**^+/-^**mice (**Figure 7C** and** 7D**). Moreover, in the footprint test, M1-TDP-43 PFFs-injected Atg5**^+/-^**mice also showed significantly shorter stride length and wider base width similar to those found in C7-TDP-43 PFFs-injected Atg5**^+/-^**mice (**Figure 7E-J**). In conclusion, these changes suggest that the M1- and C7-TDP-43 PFFs-injected Atg5^+/-^ mice exhibited ALS-like symptoms, including short survival, weight loss, abnormal gait, and motor dysfunction.

## Discussion

To investigate whether inoculation of TDP-43 PFFs can transmit pathological TDP-43 along corticospinal tract axons *in vivo*, we injected TDP-43 PFFs into the cortex or spinal cord anterior horn of Atg5^+/-^ mice. The results show that unilateral injection of TDP-43 PFFs into the fifth layer of the left M1 or the left anterior horn of C7 of Atg5^+/-^ mice could induce the formation and spreading of pathological TDP-43 along the corticospinal tract. Moreover, we also found the presence of TDP-43 pathology in our animal models at CA1 region of hippocampus and hypoglossal nucleus in M1-TDP-43 PFFs-injected Atg5^+/-^ mice at 6 mpi and in C7-TDP-43 PFFs-injected Atg5^+/-^ mice at 5 mpi. This supports the hypothesis that TDP-43 pathology could spread via axonal pathways 10. Correspondingly, the regions that TDP-43 pathology spread to are also affected in ALS patients.

It mimics the TDP-43 pathology in patients with ALS. These data indicate that TDP-43 seeding *in vivo* recapitulates ALS-like pathology. Furthermore, M1-TDP-43 PFFs-injected Atg5^+/-^ mice showed abnormal spontaneous activity in both sides of biceps brachialis, T10 paraspinals, tibialis anterior, and gastrocnemius muscles at around 5 mpi, and C7-TDP-43 PFFs-injected Atg5^+/-^ mice showed prolonged CMCT and decreased cMEP amplitude at around 2 mpi, which recapitulated the electrophysiologic changes found in ALS patients. On the other hand, these results also reflect the anterograde and retrograde spreading of pathological TDP-43.

It is known that autophagy is a vital degradation mechanism of dysfunctional organelles and large protein aggregates 21-24. Autophagy also proved to have neuroprotective effects on the nervous system as a potent inhibitor of neurodegeneration, and impaired autophagy has been evidenced in various neurodegenerative diseases including ALS 25, 26. Studies have shown that the preformed human TDP-43 aggregates were degraded via autophagy when transfected into murine NSC34 and N2a cells 27. Also, autophagy inhibition promotes the accumulation of aggregated endogenous TDP-43 and 25-kDa CTFs 28, 29. In addition, autophagy plays a critical part in TDP-43 aggregates clearance under physiological conditions 30. Under pathological conditions, *in vitro* evidence has suggested that autophagy induction improves TDP-43 metabolism and clearance and prevents death in cultured neurons and astrocytes expressing mutant TDP-43 31. Numerous studies of TDP-43 proteinopathy animal models further support the neuronal protective effects of autophagy induction. In FTLD-U mouse and *Drosophila* models for TDP-43 proteinopathy, evidence shows that inducing autophagy effectively diminishes TDP-43 aggregation and finally rescues behavior deficits 32, 33. Taken together, these studies demonstrate the relevance between autophagy and TDP-43 clearance. In our present study, we demonstrated the autophagy deficiency in M1, hippocampus, medulla oblongata, and cervical enlargement of Atg5^+/-^ mice, and pTDP-43 levels were significantly increased in M1-TDP-43 PFFs-injected Atg5^+/-^ mice compared with age-matched PBS-injected Atg5^+/-^ mice. It indicated that autophagy deficiency may promotes the formation and spreading of pathological TDP-43 *in vivo*. Furthermore, our results showed that TDP-43 PFFs-injected Atg5^+/-^ mice exhibits ALS-like neuropathology and motor phenotypes.

As we all know, SOD1 G93A transgenic mouse is a commonly used animal model for ALS. Previous studies have found increased autophagy in SOD1 G93A mice 34, 35. It would be very helpful to observe the pathological and physiological changes in SOD1 G93A transgenic mice post injection of TDP-43 PFFs. We will shift our research focus from Atg5^+/-^ mice to SOD1 G93A transgenic mice in further study, thus making our results more convincing.

In conclusion, we provided the evidence that TDP-43 PFFs could induce the transmission of pathological TDP-43 along corticospinal tract axons in both an anterograde and retrograde manner *in vivo*. These data suggest that promoting autophagy and inhibiting cell-to-cell transmission of pathological TDP-43 may have therapeutic potential in the treatment of ALS.

## Abbreviations

ALS: amyotrophic lateral sclerosis
CMCT: central motor conduction time
cMEP: cortical MEP
C7: the seventh cervical spinal cord segment
EMG: electromyography
FTLD: frontotemporal lobar degeneration
H&E: Hematoxylin and eosin
LMN: lower motor neuron
MEPs: motor evoked potentials
mpi: months post-injection
MUAPs: motor unit action potentials
M1: primary motor cortex
PFFs: preformed fibrils
pTDP-43: phosphorylated TDP-43
pTDP-43-ir: pTDP-43-immunoreactive
sMEP: spinal MEP
TDP-43: TAR DNA-binding protein-43
UMN: upper motor neuron
